# Russian‑Language Version of the Trail Making Test Implemented on the Inquisit Computer Platform

**DOI:** 10.17691/stm2026.18.4.04

**Published:** 2026-08-31

**Authors:** I.A. Fedotov, A.G. Faustova, A.A. Zaitseva, E.A. Fedotova, D.I. Shustov

**Affiliations:** MD, PhD, Associate Professor, Department of Psychiatry and Psychological Counseling; Ryazan State Medical University named after Academician I.P. Pavlov, 9 Vysokovoltnaya St., Ryazan, 390035, Russia; PhD, Head of the Department of Clinical Psychology; Ryazan State Medical University named after Academician I.P. Pavlov, 9 Vysokovoltnaya St., Ryazan, 390035, Russia; Student, Faculty of Clinical Psychology; Ryazan State Medical University named after Academician I.P. Pavlov, 9 Vysokovoltnaya St., Ryazan, 390035, Russia; Student, Faculty of Clinical Psychology; Ryazan State Medical University named after Academician I.P. Pavlov, 9 Vysokovoltnaya St., Ryazan, 390035, Russia; MD, DSc, Professor, Department of Psychiatry and Psychological Counseling; Ryazan State Medical University named after Academician I.P. Pavlov, 9 Vysokovoltnaya St., Ryazan, 390035, Russia

**Keywords:** schizophrenia, neuropsychological tests, Trail Making Test, executive functions

## Abstract

**The aim of the study** was to adapt and validate the Trail Making Test (TMT) implemented on the Inquisit computer platform.

**Materials and Methods:**

A total of 69 individuals were examined: 34 conditionally mentally healthy volunteers (12 men and 22 women; mean age 20.6 [20.1; 21.2] years) and 35 schizophrenia patients (12 men and 23 women; mean age 38.7 [34.1; 43.3] years). The test tasks and user interface of the original English‑language TMT script were translated into Russian. The correctness of the translation was verified by the Inquisit platform developers, after which the Russian‑language version was made available on the platform. The study (testing was performed twice) was conducted using the Russian‑language TMT script, the Cancellation Test, the Number Finding Test (Schulte tables), and the 10‑Word Learning Test.

**Results:**

Cronbach’s alpha coefficient was 0.96 for the conditionally mentally healthy group and 0.90 for the schizophrenia patient group, indicating sufficient internal consistency of the TMT. Quantitative measures of test‑retest reliability reached noticeable to high levels on the Chaddock scale for time‑based parameters, while no correlations were found for the number of errors. For both groups, positive statistically significant correlations were found between the time spent on the TMT and the Number Finding Test. In the schizophrenia patients, negative statistically significant correlations were found between the number of lines processed (Cancellation Test) and the time spent (TMT). These findings confirm convergent validity between TMT time scores, classical attention tests, and dynamic characteristics of thinking. At the same time, no significant correlations were found between the number of errors in the TMT and in the Cancellation Test. This might be related to different neuropsychological mechanisms underlying errors in these tests. Discriminant validity was demonstrated by the absence of significant correlations between most TMT measures and the results of the 10‑Word Learning Test in both groups.

**Conclusion:**

The adapted TMT has sufficient internal consistency, convergent and discriminant validity, and test‑retest reliability. Thus, it can be recommended for use in research to assess executive function deficits in schizophrenia patients. Notably, time spent on TMT tasks is the parameter that most closely corresponds to the assessment of dynamic attention indicators.

## Introduction

Modern research on schizophrenia has considerable attention to the neurocognitive profile, which largely determines the quality of remission [1, 2]. Objective assessment of executive functions forms the basis of the most widely used test batteries designed to detect impairments associated with schizophrenia [3, 4]. One of the most frequently used tools for assessing cognitive function is the Trail Making Test (TMT), proposed by Armitage in 1946 [5]. Initially, this test was validated in patients with local organic brain lesions, but later it became widely used in schizophrenia research as well [6–8].

The Trail Making Test consists of two parts: A and B. Parameters such as completion time for each task and the number of errors are recorded during the test. Correlation analysis has established that the time taken by a subject to complete Part A primarily reflects visual perceptual ability, while the time spent on Part B reflects working memory and attentional set‑shifting. The difference score (B–A) serves as an indicator of executive control ability [9]. In recent years, the comparability of the traditional paper‑based TMT and computerized versions has been demonstrated [10]. The computerized version of the TMT allows researchers to quickly and with millisecond accuracy assess all primary and derived measures of the test.

The Trail Making Test is being actively validated for use in psychiatry [11], addiction medicine [12], geriatrics [13], neurology [14], and other medical fields [15]. However, the use of this test in countries with alphabets other than Latin requires studies aimed at its validation and the establishment of normative values [16, 17].

**The aim of the study** was to adapt and validate the TMT implemented on the Inquisit platform.

## Materials and Methods

The study was approved by the local ethics committee of Ryazan State Medical University named after Academician I.P. Pavlov (Russia) (protocol No.1 dated September 12, 2022) and was conducted in accordance with the Declaration of Helsinki (2024).

### Research stages

At the first stage, forward and backward translations of the TMT script from the Inquisit library were performed. All tasks and the user interface of the original TMT script were translated. The correctness of the translation was verified by the developers of the Inquisit platform, after which the Russian‑language version was placed in the test library of this platform [18]. Further validation studies were conducted using the Inquisit 6.0 platform (Millisecond, USA). It is specialized software for psychological testing on a personal computer. The primary method used was the Russian-language version of the TMT script translated by the authors.

At the second stage, participants without mental disorders (respondents) were recruited by random selection for validation of the method in a group of conditionally mentally healthy volunteers. All respondents in this group had consulted psychiatrists for a medical report. In addition to completing the TMT twice on a computer in the Inquisit environment, participants performed the following psychological tests: the Cancellation Test, the Number Finding Test, and the 10‑Word Learning Test.

At the third stage, the psychodiagnostic battery described above was offered to patients with a diagnosis of schizophrenia who were receiving inpatient treatment at the N.N. Bazhenov Regional Clinical Psychiatric Hospital (Russia). The patients were selected randomly.

### Description of the Russian-language TMT script

The TMT is a method for assessing executive functions related to visual attention and the task‑switching ability. The developed script is intended for use on devices with a screen diagonal of more than 6.5 inches.

The script consists of four parts (trails): Sample A, Task A; Sample B, Task B. The total duration of all tasks does not exceed 5 min.

Sample A and Task A contain only numbers. The subject is given the following task description (Sample A): *“On this page there are several numbers. Using the mouse pointer, start with number 1 and draw a line from number 1 to number 2, then from 2 to 3, then from 3 to 4, and so on in order until you reach the end. Draw the lines as quickly as possible. To start the task, click the left mouse button on circle 1 labeled START, and then begin drawing. Make sure you hit all the circles in order”.* In case of an error, a prompt appears: the last correctly selected circle lights up yellow, and the circle to which the line should be drawn next lights up blue (see the Figure). Additionally, a text prompt is displayed, for example: *“You skipped circle 4. You have to start with number 1 and draw a line from number 1 to 2, then from 2 to 3, then from 3 to 4, and so on in order until you reach the end. Go back to circle 3 and draw a line to 4”.* Task A, which follows Sample A, contains numbers from 1 to 25 with a similar task description and prompts.

**Figure:**
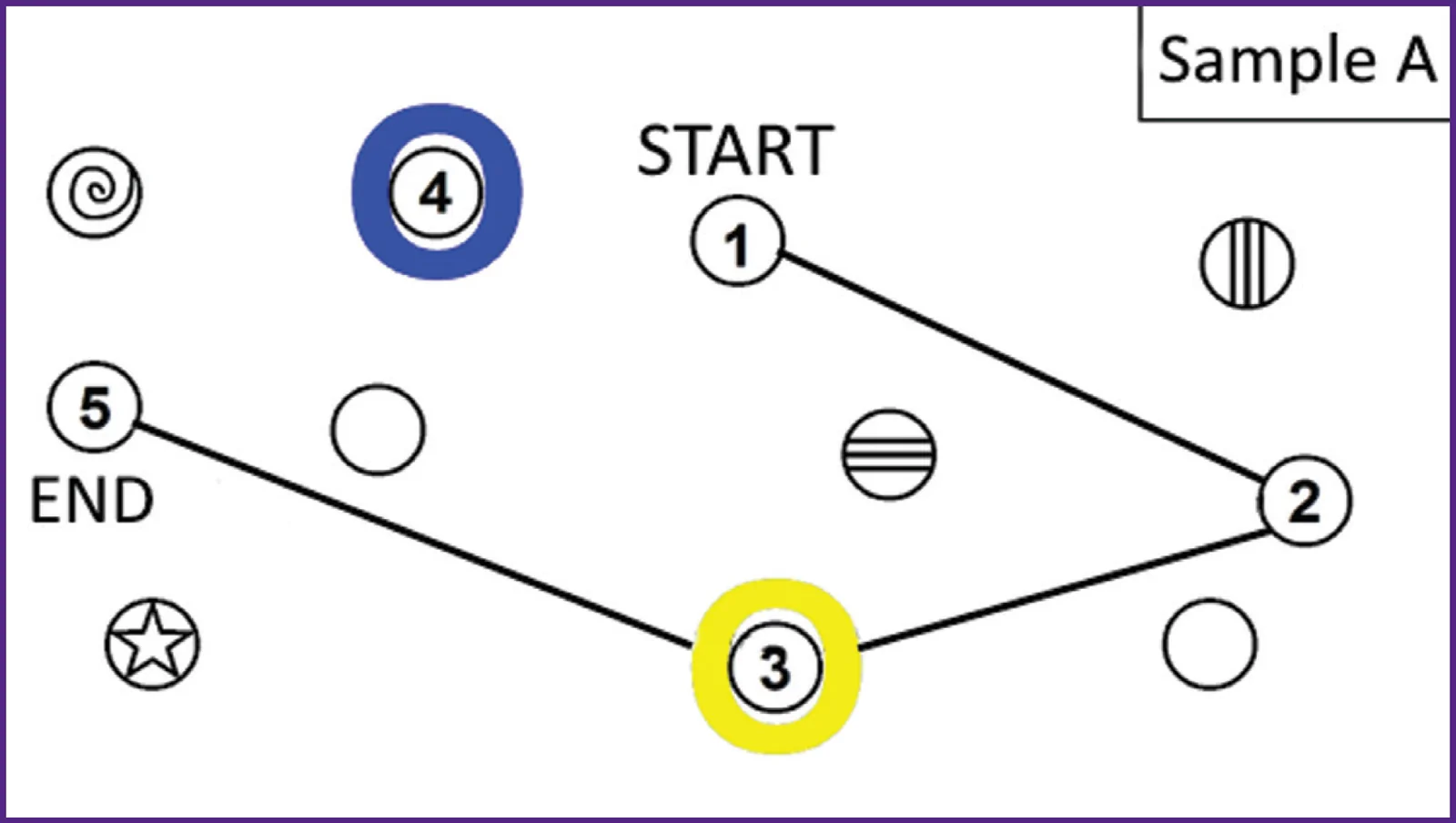
Trial display in Sample A of the Trail Making Test The subject’s task is to start moving the mouse cursor from number 1 and then connect all the remaining circles in order (2, 3, 4, 5, etc.). The subject made an error by drawing a line directly from circle 3 to circle 5. As a result, a prompt appeared: circle 3 is highlighted in yellow (where the cursor should be returned), and circle 4 is highlighted in blue (the circle to which the line should be drawn)

Sample B and Task B contain circles with numbers and letters of the Russian alphabet. The instruction for the subject is the following (Sample B): *“On this page there are several numbers and letters. Using the mouse pointer, start with number 1 and draw a line from number 1 to letter A, then from A to 2, from 2 to B, from B to 3, from 3 to C, and so on in order until you reach the circle labeled FINISH. Remember that first comes a number, then a letter, then a number, then a letter, and so on. Draw the lines as quickly as you can. To start the task, click the left mouse button on circle 1 labeled START, and then begin drawing’’.* In case of an error, a prompt is also provided.

Upon processing, the Inquisit program outputs the following TMT results:

Sample A Errors — number of errors in Sample A;
Sample A Time — time taken to complete Sample A (ms);
Sample В Errors — number of errors in Sample B;
Sample В Time — time taken to complete Sample B (ms);
Trail A Errors — number of errors in Task A;
Trail A Time — time taken to complete Task A (ms);
Trail В Errors — number of errors in Task B;
Trail В Time — time taken to complete Task B (ms);
Combined Errors — total number of errors across all four trials;
Combined Trail Time — total time spent on Task A and Task B combined (ms).

### Description of the recruited participant groups

Inclusion criteria for the study were the following: age 18 to 60 years; fluent spoken Russian; absence of mental disorders (for the control group of conditionally mentally healthy individuals); and a confirmed diagnosis of schizophrenia (F20) according to medical records (for the experimental group).

Exclusion criteria were the following: refusal to participate at any research stage; severe neurological diseases, cognitive impairments, or visual pathologies that could interfere with successful completion of the experimental tasks.

The group of conditionally mentally healthy volunteers included 34 individuals: 12 men (35.3%) and 22 women (64.7%); the mean age was 20.6 [20.1; 21.2] years. The group of schizophrenia patients included 35 individuals: 12 men (34.3%) and 23 women (65.7%); the mean age was 38.7 [34.1; 43.3] years.

Each participant underwent an interview explaining the purpose and objectives of the research. All participants provided written informed consent. One participant from the experimental group withdrew from the study during testing and was not included in the final group.

### Statistical analysis

Statistical analysis was performed using MedCalc 20.104 software (MedCalc Software Ltd, USA). Distribution normality of the obtained empirical data was assessed using the Kolmogorov–Smirnov test. Since the distribution of most variables deviated from normal, Spearman’s rank correlation was used to analyze relationships between variables.

Internal consistency of the questionnaire was assessed using Cronbach’s alpha coefficient.

Convergent and discriminant validity were assessed using Spearman’s rank correlation coefficient. The strength of correlation was qualitatively assessed using the Chaddock scale: 0.1–0.3 — weak; 0.3–0.5 — moderate; 0.5–0.7 — noticeable; 0.7–0.9 — high; 0.9–0.99 — very high. Differences were considered statistically significant at p< 0.05. Variables with non-normal distributions were presented as mean with 95% confidence intervals (CI) for continuous variables (mode) and 95% CI for discrete variables. Variables with normal distributions were presented as median with interquartile range.

## Results

### Validation of the Trail Making Test in conditionally mentally healthy respondents

To assess the internal consistency of the method, statistical analysis of scores of the Combined Trail Time, Trail A Time, and Trail B Time was performed. Cronbach’s alpha coefficient for standardized variables was 0.96, with a lower 95% CI bound of 0.93. These results confirm a sufficiently high internal consistency of the Russian‑language version of the TMT in the group of conditionally mentally healthy volunteers.

Spearman’s correlation coefficient was used to assess test‑retest reliability. The relationship between the results of the first and second TMT administrations was examined (Table 1). The interval between these sessions was approximately 40 min, during which participants completed other tasks.

**Table 1:** Correlation analysis results for the first and second Trail Making Test administrations in the group of conditionally mentally healthy respondents

Trail Making Test	r_S_	p
Combined Errors	0.32	0.0653
Combined Trail Time	0.709	< 0.0001*
Sample A Errors	−0.062	0.7256
Sample A Time	0.512	0.002*
Sample B Errors	0.202	0.252
Sample B Time	0.546	0.0008*
Trail A Errors	0.349	0.0428*
Trail A Time	0.489	0.0033*
Trail B Errors	0.225	0.2017
Trail B Time	0.737	< 0.0001*

N o t e: r_S_ — Spearman’s rank correlation coefficient; p — level of statistical significance between the first and repeated test results;

* p< 0.05.

All measured time parameters showed positive statistically significant correlations at the noticeable to high level according to the Chaddock scale. For the number of errors, a statistically significant positive correlation was found only for Task A (Trail A Errors). These findings confirm the test‑retest reliability of the TMT for temporal measures of executive function performance.

Construct validity is an important indicator of the method quality. Construct validity is most often assessed through correlation analysis with methods measuring a similar concept (convergent validity) and with fundamentally different methods (discriminant validity) [19].

To determine convergent validity, the Russian‑language version of the TMT was correlated with the results of the Cancellation Test and the Number Finding Test (Table 2).

**Table 2:** Convergent validity assessment results for the Trail Making Test in the group of conditionally mentally healthy respondents

Trail Making Test		Time to complete tables in the Number Finding Test					Cancellation Test	
		No.1	No.2	No.3	No.4	No.5	Number of errors	Number of completed lines
Combined Trail Time	r_S_	0.207	0.409	0.567	0.477	0.302	0.288	−0.107
	p	0.2407	0.0162*	0.0005*	0.0043*	0.0822	0.0987	0.5466
Sample A Time	r_S_	0.047	0.14	0.074	0.066	0.079	0.144	−0.216
	p	0.7912	0.4311	0.6765	0.7107	0.6564	0.4174	0.2198
Sample B Time	r_S_	0.33	0.355	0.675	0.565	0.404	0.214	−0.187
	p	0.0569	0.0394*	< 0.0001*	0.0005*	0.0178*	0.2248	0.2894
Trail A Time	r_S_	0.12	0.359	0.465	0.388	0.323	0.128	−0.096
	p	0.4985	0.0368*	0.0056*	0.0234*	0.062	0.4692	0.5901
Trail B Time	r_S_	0.262	0.465	0.568	0.411	0.252	0.294	0.049
	p	0.1346	0.0056*	0.0005*	0.0158*	0.1503	0.0918	0.785

N o t e: r_S_ — Spearman’s rank correlation coefficient; p — level of statistical significance between the first and repeated test results;

* p< 0.05.

Statistically significant positive correlations were found between the time to complete TMT tasks (in milliseconds) and the time spent on the Number Finding Test tables (in seconds). These results indicate convergent validity of the TMT. At the same time, no significant correlations were found between the number of errors in the TMT and the number of errors in the Cancellation Test (all p>0.05). There were also no significant correlations between the number of completed lines (Cancellation Test) and any TMT measures.

To assess discriminant validity, a correlation analysis was performed between the TMT results and the results of the 10‑Word Learning Test. No significant correlations were found between the measures of these two tests. The absence of correlations indicates discriminant validity of the TMT.

### Validation of the Trail Making Test in schizophrenia patients

To assess internal consistency of the method, statistical analysis of the Combined Trail Time, Trail A Time, and Trail B Time scores was performed. Cronbach’s alpha coefficient for standardized variables was 0.90, with a lower 95% CI bound of 0.84. These results confirm a sufficiently high internal consistency of the Russian‑language version of the TMT for the group of schizophrenia patients as well.

Spearman’s rank correlation coefficient was used to assess test‑retest reliability. The relationship between the results of the first and second TMT administrations was examined (Table 3). The interval between these sessions was approximately 40 min, during which participants completed other tasks.

**Table 3:** Correlation analysis results for the first and second Trail Making Test administrations in the group of schizophrenia patients

Trail Making Test	r_S_	p
Combined Errors	0.629	0.0001*
Combined Trail Time	0.866	< 0.0001*
Sample A Errors	−0.124	0.4778
Sample A Time	0.387	0.0216*
Sample B Errors	0.14	0.4219
Sample B Time	0.512	0.0017*
Trail A Errors	0.604	0.0001*
Trail A Time	0.697	< 0.0001*
Trail B Errors	0.36	0.0336*
Trail B Time	0.863	< 0.0001*

N o t e: r_S_ — Spearman’s rank correlation coefficient; p — level of statistical significance between the first and repeated test results;

* p< 0.05.

In the group of schizophrenia patients, as in the group of conditionally mentally healthy respondents, all time parameters correlated positively with each other. Additionally, error rates in Task A and Task B also correlated positively. Thus, the TMT demonstrated sufficient test‑retest reliability in the group of schizophrenia patients.

The results of the correlation analysis performed to assess convergent validity are presented in Table 4.

**Table 4:** Convergent validity assessment results for the Trail Making Test in the group of schizophrenia patients

Trail Making Test		Time to complete tables in the Number Finding Test					Cancellation Test	
		No.1	No.2	No.3	No.4	No.5	Number of errors	Number of completed lines
Combined Trail Time	r_S_	0.579	0.673	0.545	0.629	0.609	−0.192	−0.505
	p	0.0003*	< 0.0001*	0.0007*	0.0001*	0.0001*	0.2682	0.002*
Sample A Time	r_S_	0.095	0.255	0.192	0.298	0.091	−0.308	−0.435
	p	0.5872	0.1396	0.2691	0.0824	0.6038	0.072	0.0091*
Sample B Time	r_S_	0.456	0.382	0.359	0.427	0.493	−0.169	−0.208
	p	0.0059*	0.0235*	0.0342*	0.0105*	0.0026*	0.3327	0.2315
Trail A Time	r_S_	0.509	0.638	0.468	0.569	0.581	−0.075	−0.359
	p	0.0018*	< 0.0001*	0.0046*	0.0004*	0.0002*	0.6688	0.0342*
Trail B Time	r_S_	0.504	0.564	0.466	0.523	0.514	−0.179	−0.505
	p	0.002*	0.0004*	0.0048*	0.0013*	0.0016*	0.3045	0.002*

N o t e: r_S_ — Spearman’s rank correlation coefficient; p — level of statistical significance between the first and repeated test results;

* p< 0.05.

Statistically significant positive correlations were found between the time to complete TMT tasks and the time to complete the Number Finding Test. Statistically significant negative correlations were found between TMT time parameters and the number of lines processed by schizophrenia patients in the Cancellation Test. It means that the more time a subject spent on the TMT, the fewer lines they were able to process in the Cancellation Test. These patterns demonstrate sufficient convergent validity of the TMT. No significant correlations were found between the number of errors in the Cancellation Test and the TMT. However, schizophrenia patients had statistically significantly higher total error scores than the conditionally mentally healthy respondents (4 [2; 5] vs 1 [0; 2]; p=0.005).

To assess discriminant validity, a correlation analysis was performed between the TMT results and the scores from the 10‑Word Learning Test. The following statistically significant negative correlations were found: between the completion time in TMT Sample A and the number of words recalled after the first presentation (r_S_=−0.408; p=0.01), after the third presentation (r_S_=−0.367; p=0.02), and after the fourth presentation (r_S_=−0.367; p=0.03) in the 10‑Word Learning Test. These correlations may be related to the fact that during the first TMT task (Sample A), mechanical memory, used to retain instructions, is engaged the most. Subsequent TMT tasks predominantly reflect executive functions.

## Discussion

The TMT is quite widely used in research on executive brain functions in various countries [7]. It is also included in the approved clinical guidelines KR617_5 “Cognitive Disorders in Elderly and Senile Persons” (Appendix G13) [20]. At the same time, validation of this method on a computerized platform and in a group of schizophrenia patients allows for more active use of this approach in studies of cognitive deficits in patients with psychoses during remission.

Most researchers agree that the TMT has a complex and multifactorial structure of assessed neuropsychological parameters: visual perception, motor reaction speed, fine motor performance, visual information processing speed, mechanical memory, and formal intelligence [9]. Our results demonstrate that the time spent on Parts A and B of the TMT correlates positively with the time used to find numbers in the Number Finding Test. Therefore, it is reasonable to add “attention” to the described parameters as the most appropriate construct for correlation within the framework of a Russian neuropsychological approach. The attention parameters we studied depend on visual perception, motor reaction speed, and fine motor quality. For the schizophrenia patient group, this is also confirmed by a negative correlation with the number of completed lines (Cancellation Test). It can be assumed that these impairments will be more pronounced in patients with psychoses.

In our study, the mean completion time for Task A in the group of conditionally mentally healthy volunteers was 52.8 [48.2; 67.1] s, which is higher than the normative results for healthy respondents of the same age group in classical paper‑based testing: for European countries — 33.5 (SD=13.0) s [20], for Iran — 27.6 (SD=9.1) s [16]. The mean time for Task B was 61.1 [54.4; 76.5] s, whereas in the European group it was 78.1 (SD=33.7) s [21], and in the Iranian group — 59.8 (SD=21.6) s [16], indicating a negligible difference. Furthermore, it has been demonstrated that TMT completion time in mentally healthy respondents also depends on education level and gender; therefore, continued research is needed to obtain more differentiated normative values [21].

In our study, the mean completion time for Task A in schizophrenia patients was 96.8 [82.9; 108.1] s, and for Task B — 135.3 [112.8; 156.3] s. According to a meta‑analysis, the mean paper‑based Task A completion time in schizophrenia patients was 51.1 (SD=2.3) s, and for Task B — 126.3 (SD=10.5) s [22]. The time spent on Task A in the computerized version was nearly twice as long as in the classical paper‑based testing, while the time for Task B differed little. Apart from the influence of independent factors such as general computer literacy, computer gaming experience, and others that still need to be studied, this may be explained by a greater need for familiarization when performing the first TMT tasks; with paper and pen, this process may proceed faster.

The absence of significant correlations between the number of errors in the TMT and the analogous measure in the Cancellation Test suggests that these errors have different neuropsychological mechanisms. In the TMT, they may be related to incorrect line drawing and accidental touching of irrelevant figures. In the Cancellation Test, errors are always a sign of inattention and visual perception impairments, which are not closely related to motor function. It can be concluded that the number of errors in the computerized version of the TMT is a marker of the severity of motor disorders associated with neuroleptic therapy or catatonia (in schizophrenia patients) or general motor dexterity (in conditionally mentally healthy individuals).

The absence of significant correlations between most TMT measures and the 10‑Word Learning Test demonstrates the minor role of mechanical memory in TMT performance.

## Conclusion

The Russian‑language version of the Trail Making Test on the Inquisit platform represents a modern adaptation of a precise and widely used method for the objective assessment of cognitive abilities. This version is a valid approach for studying executive functions in both conditionally mentally healthy individuals and schizophrenia patients. Thus, it can be recommended for research and practical purposes. The time spent on task completion predominantly reflects impairments in attention.
